# Bennett Angle, Condylar and Jaw Movements in Asymptomatic Athletes with a History of a Blow to One Side of the Mandibula

**DOI:** 10.3390/dj11080195

**Published:** 2023-08-14

**Authors:** Nikolina Lešić, Davor Seifert, Dora Dragičević, Luka Pul, Dorotea Petrović, Asja Čelebić, Hrvoje Pezo

**Affiliations:** 1 Department of Dental Medicine, Faculty of Dental Medicine and Health Osijek, J.J. Strossmayer University of Osijek, 31 000 Osijek, Croatia; davor.seifert@gmail.com (D.S.); ddragicevic@fdmz.hr (D.D.); lpul@fdmz.hr (L.P.); dpetrovic@fdmz.hr (D.P.); predsjednikhsk@gmail.com (H.P.); 2 Private Dental Practice Seifert d.o.o., Martićeva ulica 43, 10000 Zagreb, Croatia; 3 School of Dental medicine, University of Zagreb, 10000 Zagreb, Croatia; celebic@sfzg.hr

**Keywords:** condylar movements, athletes, history of orofacial injuries, Bennett angle

## Abstract

Sports activities may induce long-lasting changes in mandibular trajectories. The aim was to compare condylar and mandibular movements in athletes with orofacial injuries with values measured in non-injured athletes. The group of 132 athletes without mandibular injury included asymptomatic athletes with a history of a blow to the right side (N = 43) and the group included asymptomatic athletes with a history of a blow to the left side (N = 41) of the mandible. The injured athletes suffered from stiffness/pain and/or limitation of jaw movements. The symptoms disappeared shortly after the injury. Athletes with a history of injury have smaller mean values of Bennett angle on the side of impact, and Bennett angle on the opposite side is greater than the mean found in non-injured athletes. Significantly smaller Bennett angle values in athletes with a history of a blow to one side of the mandible are due to the adaptability of the orofacial system. The larger Bennett angle on the opposite side of the injury is also due to the adaptive mechanism of the TMJ. Clinical Relevance: An individualized approach to TMJ values is mandatory in restorative procedures in every patient, especially in patients with a history of trauma to the orofacial system.

## 1. Introduction

Athletic activities have a variety of benefits for personal well-being, but recreational sports activities also have an increased risk for orofacial and/or dental injuries due to falls and collisions with players, equipment, or objects [1,2,3,4,5,6,7,8,9]. Traumatic dental and facial injuries are common in sports and often cause esthetic, functional, psychological, and environmental problems [10]. According to Clegg [11], injuries to the orofacial system account for 33% to 56% of all injuries during athletes’ careers. Dental injuries are the most common type of orofacial injuries that occur during sports participation [10,12,13]. With the increasing popularity of contact sports and the encouragement of participation at a young age, the role of dentists in the prevention of dental and other orofacial sports injuries has become very important [14]. Many orofacial injuries heal without subjective symptoms, but their consequences often persist. In prosthetic rehabilitation procedures, individual occlusal morphology and stable interocclusal contacts without any interference must be achieved. Therefore, knowledge of individual condylar movements, Bennett angle, condylar trajectory, eminence inclination, etc. is very important for proper prosthetic and gnathological rehabilitation [15]. The aim of this study was to measure condylar path, sagittal condylar path, Bennett angle, and symphyseal movements in athletes with a history of orofacial injuries and compare them with non-injured athletes. The hypothesis of this study is that the average value of the Bennett angle for the left and right temporomandibular joint, measured with an electronic device, is statistically significantly different in the control group and in athletes with macrotrauma on the left or right side.

## 2. Materials and Methods

### 2.1. Sample

The sample consisted of 132 athletes from the city of Zagreb and Zagreb County who participated in contact sports. The study included 112 men and 20 women. The average age of the respondents was 29.44 years. They were divided into three groups. The control group (A-C) consisted of 48 athletes who had never suffered a blow to the mandible. The second group included 43 asymptomatic athletes with a blow to the right side of the mandible (A-R), and the third group included 41 asymptomatic athletes with a blow to the left side of the mandible (A-L). They all had angle class I. Other participants (angle class II or III) were excluded. The target group was athletes with habitual occlusion and natural dentition. Athletes with major prosthetic procedures, bridges with more than two pontics or more than four crowns in one or more quadrants, and completely prosthetically repaired teeth are excluded from the study. Also, athletes with an abnormal or anamnestic disorder (dysfunction) are excluded from this examination, which was performed according to the RDC/TMD protocol.

### 2.2. Questionnaires

All participants completed the questionnaires that consisted of two parts. The first part of the questionnaire asked for personal data (age, sex, time of sports participation, use of an intraoral mouthguard during training and competition) and data on the types of injuries to the stomatognathic system acquired during sports activities (displaced, knocked-out, and broken teeth, pain orstiffness of facial muscles, pain when opening/closing the mouth, and temporomandibular joint injuries). The second part of the questionnaire consisted of the “Research Diagnostic Criteria for Temporomandibular Disorders” (RDC/TMD) (Croatian: DKI/TMP Diagnostic criteria for the investigation of temporomandibular disorders), which are used to diagnose and classify patients with temporomandibular disorders (TMD). Before the questionnaires were completed, the athletes received instructions and explanations about the purpose of the study. The questionnaires were completed in person with the assistance of a researcher. Signed informed consent was obtained from each participant. The college ethics committee approved the study.

### 2.3. Recording of the Movements of the Mandible and Condyles

The movements of the two condyles and the mandible were recorded with the ARCUSdigma II ultrasound scanner (KaVO, Bieberach an den Ries, Germany).

Before the recordings were made with the ARCUSdigma II ultrasound scanner, impressions of the maxillary dental arches and the mandibular dental arches were taken from each participant using irreversible hydrocolloid alginate impression material (Aroma Fine Plus, GC, Tokyo, Japan). The dental impressions were cast in a plaster material type IV (Fuji Rock, GC Leuven, Belgium). According to the manufacturer’s instructions, the individual paraocclusal trays were matched to the plaster casts using a cold-curing acrylic resin (Ostron, GC, Leuven, Belgium). One paracclusal tray was firmly fixed to the lower teeth with an acrylic resin for temporary restorations (Structur, Voco, Cuxhaven, Germany). The paraclusal tray or the acrylic resin used to secure the paraclusal tray did not affect the maxillary teeth either in the maximum intercuspation position or during jaw movements.

Images were taken using the ARCUSdigma II six-degree-of-freedom jaw-tracking ultrasound scanner. Each athlete sat comfortably on a chair (upright posture). A transmitter of the recording device was attached to the mandible using a paraocclusal cup, and the sensors were attached to the participant’s head using a facebow. The recording device measured in real time the latency between transmitted and received ultrasound pulses.

Based on the concept of six degrees of freedom, the software of the device calculated the spatial position of the condyles, i.e., in the present study, the Bennett angles of the right and left condyle.

All condylar movements were registered without the influence of occlusion. The measurements were performed as follows: A palatal plate was first constructed with a pin on each participant’s maxillary model and then palatally attached to the maxillary teeth. For the mandibular casts, a metal plate was embedded in a self-curing acrylic. On both jaws, both an acrylic plate with a post and a metal plate were embedded in an acrylic resin. The post was adjusted to achieve minimal disclusion of the tooth contacts. Minimal disclusion means that there were no antagonistic tooth contacts in the centric relation position or during other mandibular movements. In each participating group (A-C, A-R and A-L), the Bennett angles, the length of the mediotrusive condylar paths, and the length of the symphysis movements (contact point between the left and right mandibular central incisors) to the left and right sides were measured.

### 2.4. Statistical Analysis

Statistics were performed using SPSS for Windows 22 software (IBM Corp., Armonk, NY, USA). It included descriptive statistics (means and standard deviations), Student’s *t*-test, and one-way analysis ANO-VA (Sheffe post hoc) with a significance value of 0.05.

## 3. Results

**Figure 1:**
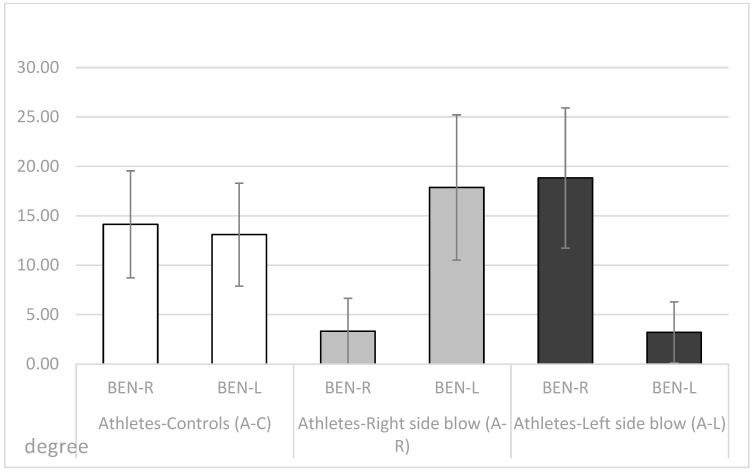
Means and standard deviations for the right (BEN-R) and the left Bennett angle (BEN-L) in the A-C, the A-R and the A-L group.

**Figure 2:**
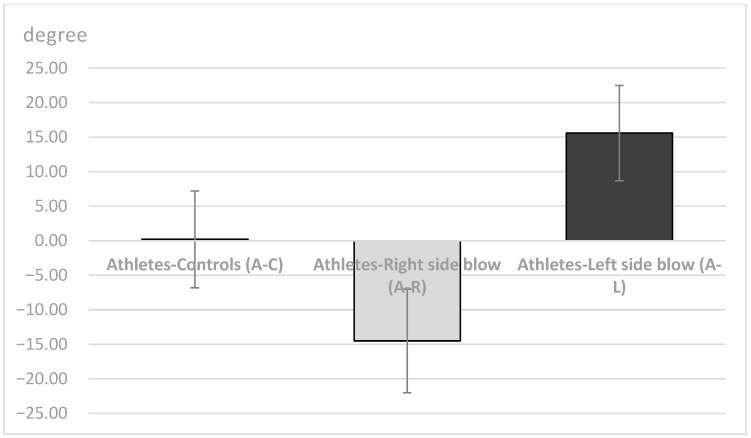
Difference (mean values and standard deviations) between the right (BEN-R) and the left Bennett angle (BEN-L) in the A-C, A-R and the A-L groups.

Descriptive statistics (means, SDs, minimum, maximum and range) of the obtained Bennett left (BEN-L) and right (BEN-R) angles and the difference (DIFF) between BEN-R and BEN-L are shown in Table 1, Figure 1 and Figure 2 for all three observed groups.

The control group of athletes (A-C) had similar left and right Bennett angles with a very small mean difference (0.52°). The athletes in the group who reported a blow to the right side of the mandible (A-R) had smaller mean values of the Bennett angle of the right condyle (BEN-R) when the condyle movement was to the left. Fourteen of them showed a Bennett angle of the right condyle of 0° and 27 athletes showed an angle of 4°. The measured Bennett angle of the right condyle deviated from the above values only in two of the injured athletes, which were greater than 14°. While their left Bennett angle had much higher values (BEN-L). The athletes in the group (A-L) who reported a blow to the left side of the mandible had smaller Bennett angle mean values of the left condyle (BEN-L) during mediotrusive condylar movement to the right side. Thirteen of them showed a Bennett angle of the left condyle of 0° and 26 athletes showed an angle of 4°. The Bennett angle of the left condyle deviated from the above values only in two of the injured athletes, which were greater than 13°. Similar to the (A-R) group, the (A-L) group also showed higher mean values of Bennett angle on the opposite side of the injury (BEN-R).

The unilateral ANOVA showed significant differences between the mean values of the 3 groups (A-C, A-R, A-L; DF1 = 2, DF2 = 129); F = 89.95, *p* < 0.001 for the right Bennett angle (BEN-R); for the left Bennett angle (BEN-L; F = 78.34, *p* < 0.001), and for the difference between the right and left Bennett angles (F = 239.94, *p* < 0.001). Post-hoc Sheffe tests showed significant differences between the 3 observed groups for all three variables (*p* < 0.05; BEN-R, BEN-Land DIFF).

The results showed that both groups who received a blow to the face had significantly smaller Bennett angle on the side of a blow during mediotrusive condylar movements (Table 2). Further, the A-R group and the A-L group had significantly smaller values of the Bennet angle on the side of a blow than of the contralateral side, while the group A-C had no significant difference between the left and the right side (Table 2).

**Figure 3:**
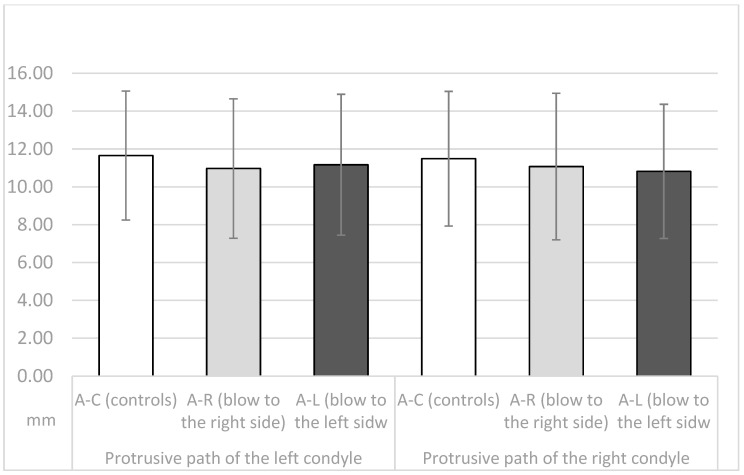
Length of the protrusive path of the right and the left condyle in the A-C, the A-R and the A-L group (means and standard deviations).

The length of the protrusion path of the right and left condyle in the A-C, A-R and A-L groups (means and standard deviations) is shown in Figure 3. There was no significant difference between the 3 groups for the length of the protrusion path of the right condyle (unilateral ANOVA, F = 0.382, *p* = 0.68, NS), the left condyle (unilateral ANOVA, F = 0.442, *p* = 0.644, NS), or for the difference of the protrusion paths between the right and left condyle (unilateral ANOVA, F = 0.46, *p* = 0.96).

**Figure 4:**
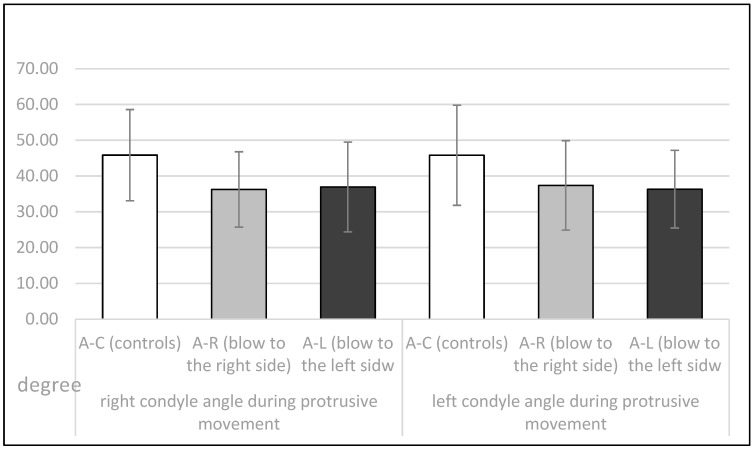
Sagittal condylar path (in degrees) during the protrusive movements for the right and the left condyle in the A-C, A-R and the A-L group.

The mean values and standard deviations of sagittal condylar path (in degrees) during protrusive movements of the right and left condyle in the 3 respective groups (A-C, A-R and A-L) are shown in Figure 4. The right condyle had a significantly steeper angle in the A-C group than in the A-R and A-L groups (unilateral ANOVA; F = 9.1, *p* < 0.01), as did the left condyle (unilateral ANOVA; F = 7.77, *p* < 0.01). The post-hoc Sheffe test showed a significantly steeper angle of the sagittal condylar pathway in the A-C group than in the A-R and A-L groups (*p* < 0.05).

**Figure 5:**
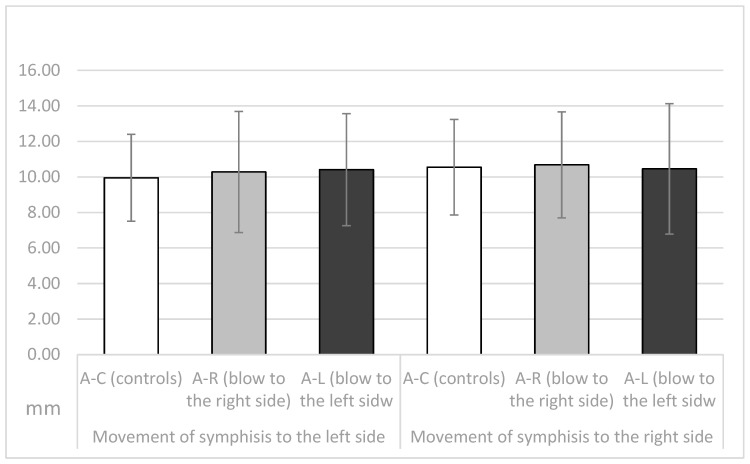
Movemets of the symphisis (in mm) during the laterotrusive movements in the A-C, the A-R and the A-L group.

The movements of the symphysis to the left and right sides in the three observed groups are shown in Figure 5. There were no significant differences in symphysis movement to the right side (F = 0.57, *p* = 0.945) or to the left side (F = 0.278, *p* = 0.758) between the three observed groups.

**Figure 6:**
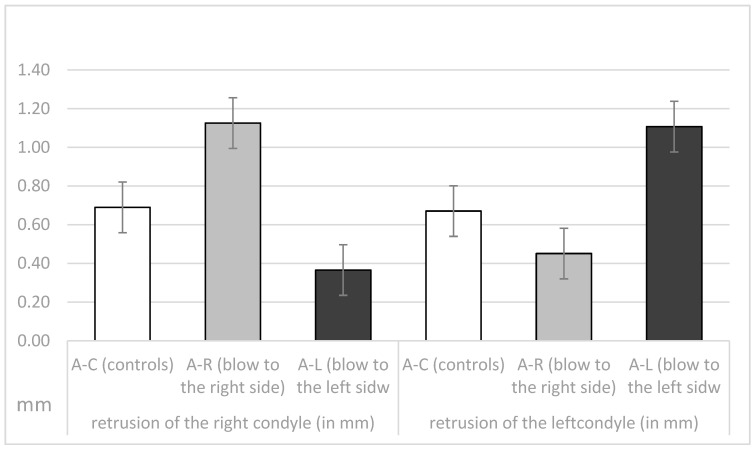
Length of the retrusion (in mm) of the right and the left condyle in the A-C, the A-R and the A-L groups.

The length of the retrusive movement of the right and the left condyle (in mm) in the three observed athlete groups is presented in Figure 6. The control group showed a similar length of retrusion between the left and right condyle. The groups studied with trauma showed greater movement on the condyles where the trauma occurred than on the contralateral side.

**Figure 7:**
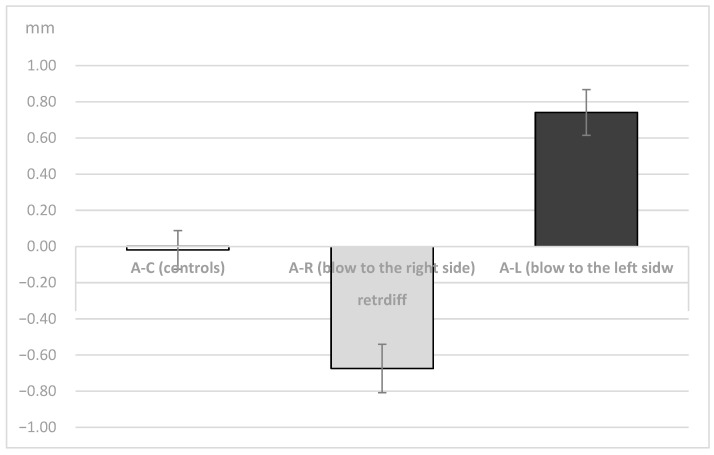
Difference of the length of the retrusive movement (in mm) between the right and the left condyle in the A-C, A-R and the A-L groups.

The difference between the right and the left condyle’s retrusive movement in the A-C, the A-R and the A-L groups is presented in Figure 7. The results collected show no significant difference between retrusive movements in the control group, while the studied groups have values of −0.67 and 0.74.

Unilateral ANOVA showed significant differences between the means of the 3 groups (A-C, A-R, A-L; DF1 = 2, DF2 = 129); F = 16.47, *p* < 0.001 for right condyle retrusion (in mm); for left condyle retrusion (F = 11.57, *p* < 0.001) in mm; and for the difference between the right and left condyle (F = 32.26, *p* < 0.001). Post-hoc Sheffe tests showed significant differences between the 3 groups (*p* < 0.05).

The significant difference between the right and left condylar restriction motion (in mm) in groups A-C, A-R and A-L, assessed by Student’s *t*-test for dependent samples, is shown in Table 3. The groups that had received a blow (A-R and A-L) showed a significant difference between the left and right sides, whereas this was not the case for the A-C group.

## 4. Discussion

Dental and facial injuries are still a common problem among athletes in team sports. Facial injuries are possible in anyone, although they are more likely in those who have more physical contact. Fractures, intrusions, extrusions, oral avulsions and temporomandibular joint dislocations are common dental injuries in sports [16]. 

Athletes who play contact sports sometimes receive a blow to the mandible. The short duration of pain and other disturbances following such a blow usually disappear within a few days, and athletes may be symptom-free thereafter. However, a blow may have adverse effects on the fibrous or cartilaginous tissue in the temporomandibular joint, and the consequences may be evident long after the trauma [17,18,19,20,21]. The majority of hits to the jaw don’t cause fractures. The temporomandibular disc and its supporting components may sustain serious damage if large pressures are applied to them. The retrodiscal tissues may be compressed if the condyle is pushed posteriorly. Trauma to the TMJ can occasionally result in intracapsular hemorrhage, which causes the joint to become ankylosed [22,23]. 

Therefore, in this study, some parameters of TMJ movements were measured in athletes who had never received a blow to the mandible and in athletes who had received a blow to the left or right side of the mandible in the past. All were asymptomatic at the time of recordings, with no pain or movement abnormalities. At the time the data were recorded, all athletes had a period of at least two years from the time of injury. The injured athletes experienced stiffness/pain of the masseter muscles, pain in the temporomandibular joints (preauricular pain), and/or limitations in jaw movements immediately after the injury. Symptoms continued to occur for several days afterward. In addition, some participants reported dental injuries, lacerations, contusions, and erosions of the soft tissues after a blow to the mandible. However, all symptoms diminished and eventually disappeared several days or weeks after the injury. At the time of this study, all athletes showed no subjective symptoms of temporomandibular dysfunction according to the RDC/TMD protocol and were completely satisfied with the function of their orofacial system. The length of the protrusive condylar pathway and symphysis movements were almost the same in all observed groups (*p* > 0.05, Figure 3 and Figure 5). However, the Bennett angle was significantly smaller on the side of the received blow than on the contralateral side. It was also significantly smaller than in the A-C group. The retrusive motion of the condyle on the side of the received blow was significantly longer than that of the contralateral condyle and vice versa. The sagittal condylar path was significantly higher in the A-C group, which could be a coincidental result because all angular values correspond to the range reported in the dental literature [24,25,26,27,28]. Our main results showed a significantly smaller Bennett angle on the side of the received blow during mediotrusive condylar movements (*p* < 0.01). This can be attributed to the process of adjusting the movements within the structures of the temporomandibular joint as a result of a blow. This was probably compensated by the rotational movement of the contralateral condyle or by retrusive movements, as the movements of the symphysis to the side of the blow or to the contralateral side remained almost the same [15,29,30,31,32,33,34,35]. However, there is a large difference in the average value of the Bennett angle in athletes with macrotrauma on the right or left side. In athletes who suffered trauma on the right side, the difference in the average value of the Bennett angle is 14.55°, and the difference in the average values is Bennett angle in trauma on the left side is 15.61°. Limitation of the right Bennett angle is also registered in athletes who suffered trauma on the right side, while the left.

Bennett angle in the same athletes is about 5° higher than the same angle in the control group. A restriction of the left Bennett angle was registered in athletes who suffered a blow to the left side, while the right angle in the same athletes is greater than the average Bennett angle in the control group groups. The patient’s age and the TMJ and surrounding structures’ developmental stage are important considerations in the treatment of any injury. Malformations may result from improper treatment or a failure to diagnose [36,37]. Because all patients require an individualized approach to reconstructive surgery, these data may be helpful. However, further studies on a larger group are needed to explain more precisely what happens within the structures of the temporomandibular joint after the blow. 

Even though the ARCUSdigma allows the registration of mandibular and condylar movements, the movements that can be recorded and reproduced are stereotypical and do not reflect the dynamics of functional movements. Since the 1990s, there has been a growing interest in overcoming those limitations with jaw robots. Carossa et al. (2020) present in their article system named Bionic Jaw Motion (BMJ; Bionic Technology, Vercelli, Italy) that analyzes jaw movement and can be used as a robotic In contrast to conventional systems that use pantographs and individual articulators, BJM quickly records and replicates individual mandibular movements. To prevent possible mistakes in the clinical identification of extraoral landmarks, which are practically impossible to identify without ambiguity, an intraoral reference system is implemented. In addition to boundary movements, BJM also enables the recording of functional motions. It might also be helpful for researching and identifying temporomandibular problems [38].

## 5. Conclusions

Asymptomatic athletes who had suffered a blow to one side of the mandible in the past had significantly smaller mean values of the Bennett angle on the side of the blow during condylar mediotrusive movements, suggesting a permanent change in the TMJ functional pathways after the blow. The compensatory Bennett angle on the opposite side of the injury is greater than average. The revealed change in Bennett angles in athletes with a blow to the mandible must lead to individualised measurement of jaw motion in prosthetic therapy. When athletes with a history of macrotrauma to the orofacial system require prosthetic therapy, iatrogenic interference will occur without individualised adjustment of the articulator. This interference can lead to temporomandibular dysfunction over time. All patients require an individualised approach to their orofacial system during reconstructive procedures, and this is the only way to avoid additional damage to the system.

However, further studies on a larger group are needed to explain more precisely what happens in the structures of the temporomandibular joint after the blow.

## Figures and Tables

**Table 1:** Means, SDs, minimum, maximum, and range of the left (BEN-L) and right (BEN-R) Bennett angles, and the difference (DIFF) between the BEN-R and the BEN-L.

Observed Group	Variable	Mean Angle (x in Degrees)	Standard Deviation (SD)	Minimum–Maximum	Range	95% Confidence Interval for Mean
Athletes- Controls (A-C) (N = 48)	BEN-R	14.13°	5.41°	5.80–25.8°	20.00°	12.56–15.7°
	BEN-L	13.61°	5.21°	5.80–25.8°	20.00°	12.1–15.13°
	DIFF	0.52°	4.52°	−8.5–8.5°	17.00°	−0.79–1.83°
Athletes- Right side blow (A-R) (N = 43)	BEN-R	3.33°	3.33°	0.00–16.6°	16.60°	2.31–4.35°
	BEN-L	17.87°	7.34°	3.80–30.0°	26.20°	15.61–20.13°
	DIFF	−14.54°	7.30°	0.20–27.6°	27.40°	−16.59–12.29°
Athletes- Left side blow (A-L) (N = 41)	BEN-R	18.83°	7.09°	7.20–30.0°	22.80°	15.59–21.06°
	BEN-L	3.21°	3.08°	0.00–14.7°	14.70°	2.24–4.19°
	DIFF	−15.62°	6.95°	3.20–27.6°	24.40°	13.42–17.8°

N—number of athletes, BEN-R—Bennett angle on the right side, BEN-L—Bennett angle on the left side, DIFF—difference between the left and the right Bennett angle, Mean—arithmetic mean, Minimum—the smallest achieved result, Maximum—the highest achieved result, Range—range of results, SD—standard deviation.

**Table 2:** Significance of the difference between the right and the left Bennett angle in the A-C, A-R and the A-L groups assessed by Student *t*-test for dependent samples.

Group	Difference BEN-R-BEN-L	SD of the Difference	t	df	*p*
A-C Athletes-Controls	0.52	4.52	0.79	47	0.43 NS
A-R Athletes—blow to the right side	−14.54	7.3	−13.06	42	<0.001 **
A-L Athletes—blow to the left side	15.61	6.95	14.39	40	<0.001 **

** Statistically significat with an error of less than 1%. BEN-R = Bennett angle of the right condyle; BEN-L = Bennett angle of the left condyle; t = t value, df = degrees of freedom; *p* = *p* value; NS = non statistically significant.

**Table 3:** Significance of the difference between the right and the left condyle retrusion (in mm) in the A-C, A-R and the A-L groups assessed by Student *t*-test for dependent samples.

Group	Difference: Retrusion of the Right Condyle-Retrusion of the Left Condyle	SD of the Difference	t	df	*p*
A-C Athletes-Controls	0.018	0.74	0.18	47	0.861 NS
A-R Athletes—blow to the right side	−0.67	0.88	5.05	42	<0.001 **
A-L Athletes—blow to the left side	0.74	0.83	−5.86	40	<0.001 **

** Statistically significat with an error of less than 1%. t = t value, df = degrees of freedom; *p* = *p* value; NS = statistically significant.

## Data Availability

Data sharing is not applicable to this article.
